# Editorial: Insights in cardiovascular imaging: 2021

**DOI:** 10.3389/fcvm.2022.1061337

**Published:** 2023-01-04

**Authors:** Ramya Parasa, Karl J. Weiss, Christos V. Bourantas, Steffen E. Petersen, Sebastian Kelle, Ross J. Thomson

**Affiliations:** ^1^Barts Heart Centre, St Bartholomew's Hospital, Barts Health NHS Trust, London, United Kingdom; ^2^William Harvey Research Institute, NIHR Barts Biomedical Research Centre, Queen Mary University London, London, United Kingdom; ^3^Department of Internal Medicine and Cardiology, German Heart Institute Berlin (DHZB), Berlin, Germany; ^4^German Centre for Cardiovascular Research (DZHK), Partner Site Berlin, Berlin, Germany; ^5^Institute of Cardiovascular Sciences, University College London, London, United Kingdom

**Keywords:** echocardiography, computed tomography coronary angiography, cardiac magnetic resonance imaging, strain imaging, plaque characterization, machine learning

Imaging plays an increasingly important role in the diagnosis of cardiovascular disease and the understanding of cardiovascular physiology, driven by technological advancements, integration into clinical practice, and increasing evidence of clinical effectiveness. Here we highlight a few of the many important studies that were published under the topic “*Insights in Cardiovascular Imaging: 2021*.”

## Echocardiography

Enabling diagnosis of cardiac conditions with widespread availability and minimal side effects, echocardiography is often the primary cardiac imaging modality. There has been remarkable advancement recently in long-established features of echocardiography such as tissue Doppler imaging, contrast echocardiography, myocardial speckle tracking, and real-time 3-D echocardiography.

The right atrium (RA) has an important role in the function of the right heart, with its triple function as a reservoir receiving blood from the venae cavae during ventricular systole, a conduit transferring blood to the right ventricle during early diastole, and with its active contractile function pushing blood into the right ventricle during late diastole. For a long time, the right atrium has received little notice, but has recently been the focus of numerous studies.

Lagrangian strain (ε) is the change in myocardial fiber length from diastole to systole, as a percentage of the diastolic length, and strain rate is the change of strain per unit time (1). Strain can be assessed by echocardiography using algorithms to track the movement of speckles—natural contrast features in the myocardium generated by ultrasound scatter and interference. This functionality is similar to cardiac magnetic resonance imaging feature tracking (CMR FT), where an analogous approach is used to estimate strain using natural contrast features in steady state free precession (SSFP) cine images. Speckle tracking echo has been shown to have comparable performance to CMR FT (2).

The systematic review and meta-analysis by Hosseinsabet et al. provides new insights into normal RA phasic function. Data from 15 studies involving 2,469 healthy subjects were combined to derive normal ranges for RA strain during the reservoir, conduit and contraction phases. The authors commented on sources of inter-study heterogeneity, including technical factors such as the software used for speckle tracking and the method used to derive a global value, and biological factors such as the body mass indices and body surface areas of the participants. This study provides important information to contextualize future measurements of RA function in different disease states, and highlights the importance of working to standardize RA strain measurement.

Another study by Richter et al., throws light on RA function in 56 patients being treated for pulmonary arterial hypertension (PAH). PAH is known to be associated with impaired RA reservoir and conduit function, making it important to understand how these parameters vary with treatment (3). Change in peak longitudinal strain (PLS) during follow-up, reflecting a change in RA reservoir function, was independently associated with a change in echocardiographic parameters of right heart function, including TAPSE, RV end-systolic area, and RV global longitudinal strain. Patients with increased RA reservoir function during follow-up had a reduced pulmonary artery systolic pressure and pulmonary vascular resistance as determined by right heart catheterization. On the contrary, passive strain (reflecting RA conduit function) and peak active contraction strain (representing RA contraction) showed no correspondence with invasive measurements. It is interesting to note that the RA functional patterns were associated with clinical outcomes, with worsening RA PLS associated with clinical deterioration (hazard ratio for hospitalization or death 4.87; 95% CI: 1.26–18.76; *p* = 0.022) and poor prognosis as compared to those with stable or improved RA PLS (log-rank *p* = 0.012).

## Computed tomography

Computed tomographic coronary angiography (CTCA) is a well-established non-invasive imaging modality, which allows assessment of plaque composition and burden, including plaque characteristics associated with increased vulnerability. In addition, CTCA-based coronary artery reconstruction enables the assessment of local hemodynamic forces, which in turn regulate plaque formation and allow the prediction of lesions that are prone to progress and cause events.

Quantifying high-risk plaque burden assists in the prevention of future coronary events (4). Low attenuation coronary plaque (LAP) volume >4% on CTCA is known to be a predictor of cardiovascular events, including death, at 5 years (5). A study investigating the association of low attenuation coronary plaque burden and epicardial adipose tissue volume with adverse cardiovascular events highlights the importance of CTCA in assessing plaque burden and plaque characterization (Yamaura et al.). 375 patients with suspected stable angina but no previous history of MI, PCI or CABG were included. At a mean follow-up of 2.2 years, the primary endpoint of death, non-fatal MI or urgent revascularization was observed in 15 patients (4%), of whom 65% were from the fourth quartile of LAP volume. LAP volume independently predicted the occurrence of the primary outcome after adjustment for CAD-RADS score (a grading system based on the maximum severity of obstruction in any coronary artery), coronary artery calcium score (CACS) > 100, and Suita score (a cardiovascular risk prediction score used in Japan). CACS and epicardial adipose tissue volume were associated with LAP volume. This study emphasizes the importance of factors other than stenosis severity in determining and predicting the risk of future events, and should encourage prospective research in larger samples to clarify the role of LAP volume in clinical practice.

CTCA reliably identifies coronary artery plaque with high-risk features such as low attenuation, napkin-ring-associated lesions, or positive remodeling (6). Abnormal fractional flow reserve derived from CTCA (CT-FFR) has been attributed to larger plaque burden, positive remodeling and low attenuation plaque on the CTCA, in the absence of an anatomically large plaque (7). The prospective, observational, multicenter registry China CT-FFR Study-2 aims to investigate plaque anatomy and physiology in patients undergoing CTCA for the evaluation of known or suspected coronary artery disease. Around 10,000 participants who are found to have non-obstructive coronary disease will be followed up for major adverse cardiovascular events, to determine the performance of various CT-based measures in determining outcome (Zhou et al.). It is promising further insight into the potential role of CTCA as a screening tool in outpatient settings and may throw light on the plaque pathophysiology in patients with mild to moderate coronary disease, as most cardiovascular events occur in this group of patients.

## Cardiac magnetic resonance imaging

Cardiac magnetic resonance imaging (CMR) has become an increasingly important tool for both functional assessment and tissue characterization. For functional analysis, identifying sub-clinical stages of LV systolic dysfunction is a promising role for CMR.

Mandry et al. studied 118 participants free from established cardiovascular disease, but with a high prevalence of risk factors (26% had hypertension and 52% were obese). Subjects underwent ejection fraction (EF) assessment using CMR, with phase-contrast sequences to determine aortic flow and therefore cardiac output, at baseline and median follow-up of 5.2 years. Change in EF between baseline and follow-up was inversely related to change in mean systolic (*r*_s_ = −0.02, *p* = 0.030) and diastolic blood pressure (*r*_s_ = −0.02, *p* = 0.027) and to a greater extent to change in SVR (r_s_ = −0.44, *p* < 0.001), indicating that LV afterload affects EF. The findings of this study highlight the importance of recording the conditions under which EF is measured, and the potential role of this confounder when assessing serial changes in EF.

In a similar vein, Fischer et al. provided insight into how coronary autoregulation copes with changes in blood pressure in the intra or perioperative state. They used vasoactive drugs to manipulate the mean arterial blood pressure (MAP) in 10 anesthetized pigs, in steps of 10–15 mmHg from 29 mmHg to 196 mmHg, inside an MRI scanner. Oxygen-sensitive cine images were acquired in each hemodynamic state, and LV and RV circumferential systolic and diastolic strain were measured using CMR feature tracking software. Coronary blood flow was measured using a surgically implanted Doppler ultrasound probe, and the “autoregulatory zone,” the range of MAP in which coronary blood flow remains relatively constant, was calculated for each animal. LV and RV peak strain were compromised when MAP was both below and above the autoregulatory zone. Time to peak strain was higher, and strain rate was lower, when MAP was above the autoregulatory zone. LV peak strain was linearly and inversely related to myocardial oxygenation, determined using oxygen-sensitive CMR images, when MAP was below the autoregulation zone.

Mass immunization has helped mitigate the burden of the COVID-19 pandemic on healthcare systems across the world. Myocarditis has been reported as a consequence of both COVID-19 infection and vaccination (8). Jahnke et al. report four cases of myocarditis soon after COVID-19 vaccination in young men who presented with symptoms of chest pain, fatigue and dyspnea within 3 days of vaccination, and who were found to have elevated cardiac enzymes (9). After ruling out coronary artery disease by coronary angiography and structural abnormalities by echo, CMR identified acute inflammation in T2-weighted images, and scar in late gadolinium enhancement images. Patients were subsequently treated with anti-inflammatory therapy and supportive management. This case series highlights the role of CMR in identifying myocardial inflammation and guiding management.

Laohabut et al. investigated myocardial extracellular volume (ECV) by CMR T1 mapping in 739 patients with and without type II diabetes mellitus (T2DM) who were undergoing investigation for known or suspected ischemic heart disease. Compared to patients without T2DM, those with T2DM had higher ECV and were more likely to have late gadolinium enhancement, but there was no difference in ejection fraction. ECV was independently associated with increased hazard of adverse cardiovascular outcomes (HR: 2.01, 95% CI: 1.03–3.93) and provided improved prognostic performance when added to a model including typical cardiovascular risk factors, diagnosis of T2DM, and LV ejection fraction.

Amyloid A (AA) amyloidosis is a rare condition characterized by the deposition of fibrils of the acute phase protein serum amyloid A in tissue. In contrast to transthyretin and light chain (AL) amyloidosis, cardiac involvement is uncommon and not well characterized. Chamling et al. report the CMR findings in two patients with suspected cardiac involvement in AA amyloidosis that was subsequently confirmed by endomyocardial biopsy. Both patients were found to have markedly elevated native T1 and ECV, similar to other forms of cardiac amyloidosis, but without the typical presence of late gadolinium enhancement. This study provides valuable insight into the CMR findings in cardiac AA amyloidosis, and will be of value to clinicians presented with this rare condition.

Machine learning (ML) algorithms incorporated into imaging technologies promise to further improve time efficiency and reproducibility of CMR. Rauseo et al. published a paper where they compared manually contoured datasets of short axis cine CMR images by expert analysts with automated contours derived using an ML algorithm. The results showed that automated contours scored better than the manual contours (OR 1.17, 95% CI 1.07–1.28, *p* = 0.001; *n* = 9401) when presented to blinded experts.

Numerous algorithms have been developed to automatically segment the myocardium by defining endo- and epicardial contours, with the goal of saving clinicians time and improving accuracy and reproducibility. Similarly, automatic myocardial segmentation using a machine learning technique has been explored by Chen et al. to improve the tedious and time consuming manual myocardial segmentation of MRI sequences. The team proposed a two-stage segmentation framework in which conventional neural networks and recurrent neural networks were combined, incorporating temporal information which demonstrated a 2% improvement in the Dice coefficient on the automated cardiac diagnosis.

In summary, these advancements in cardiovascular imaging hold promising new perspectives for echocardiography, computed tomography and cardiac magnetic resonance imaging. They further support the paradigm shift toward non-invasive assessment for patients with established or suspected cardiovascular disease. Further research is needed to fully assess their true value in everyday clinical practice.

## Author contributions

CB, SP, and SK contributed to conception and design of the editorial. RP, KW, and RT drafted and revised the manuscript. All authors contributed to manuscript revision, read and approved the submitted version.
